# Characterization of Glycoproteins of Native 19kDa C-Terminal Merozoite Surface Protein-1 from Native Antigen of *Plasmodium falciparum*

**Published:** 2019-09-30

**Authors:** Sahar Tajik, Sedigheh Sadeghi, Ayda Iravani, Mitra Khalili, Mohammad Arjmand, Nassir-Ud Din, Farideh Vahabi, Hossein Feiz-Haddad, Behzad Lame-Rad, Saied Reza Naddaf, Zahra Zamani

**Affiliations:** 1 Department of Biochemistry, Payame-Noor University, Tehran, Iran; 2 Department of Biochemistry, Pasteur Institute of Iran, Tehran, Iran; 3 Institute of Bioinformatics, Lahore, Pakistan; 4 Department of Parasitology, Ahvaz Jundishapur University of Medical Sciences, Ahvaz, Iran; 5 Department of Parasitology, Pasteur Institute of Iran, Tehran, Iran

**Keywords:** Merozoite surface protein1, C-terminal 19kDa, *Plasmodium falciparum*, Glycoproteins

## Abstract

**Background:**

*Plasmodium falciparum* is the protozoan parasite which causes malignant malaria of medical concern. Prime candidates for recombinant vaccine development are asexual stage antigens of *P. falciparum*, for example, merozoite surface proteins (MSP1 and MSP2) not given satisfactory results to date. In this study, the 19kDa C-terminal of MSP1, a vaccine candidate was purified in its native form in the ring stage, and its glycoproteins studied.

**Methods:**

The study was carried out at the Biochemistry Department of Pasteur Institute of Iran in the years 2015–2016. Large scale culture of *P. falciparum* was performed *in vitro* with 80% ring stage parasitemia. Isopycnic ultracentrifugation with 36% sucrose and analytical SDS-PAGE on the supernatant and precipitate performed, and the 19kDa antigen was obtained by cutting it from strips of preparative SDS gels. Purified protein was concentrated and analyzed by SDS-PAGE and immunoblotting, using antibodies raised to recombinant C-terminal MSP1.

**Results:**

The purified protein gave a single band of 19kDa antigen as shown by silver staining of SDS-PAGE and a single bond in immunoblotting. Bioinformatics also confirmed the likelihood of the presence of glycans on the antigen.

**Conclusion:**

The presence of N and O-glycoproteins were detected by Q proteome kit. This work was done on the ring stage, and earlier workers confirmed the presence of glycoproteins on MSP1 in the other stages. This glycosylation is present in all stages, and maybe incomplete protection elicited by recombinant MSP1 antigens is due to lack of N and O-glycoproteins.

## Introduction

*Plasmodium falciparum* is one of the causative agents of human malaria, a disease which accounts for almost 900000 deaths per year, the significant portion of whom are children and pregnant women in developing countries according to the Malaria fact sheet (1). Today, many investigations aim to achieve more efficient medications to conquer drug-resistant malaria parasites or to find new candidate proteins to develop vaccines for disease prevention in endemic areas (2). The biology of *P. falciparum* has been studied to figure out its interactions with the host. In all stages of the parasite life cycle, carbohydrate-recognizing proteins play a major role in virulence of the disease and its severity (3). Current vaccine approaches focus on recombinant proteins or synthetic peptides of the asexual stage. Some synthetic peptides have reached the clinical trials stae and seem to be promising, but until today, the lack of success with recombinant vaccines could be due to lack of their glycosylation (4).

Vaccine research on blood-stage malaria has concentrated on antigens expressed on the surface of merozoites. Red blood cells (RBCs) are ruptured, releasing merozoites that quickly invade other RBCs. The specific antibodies raised against merozoite surface proteins (MSPs) have only a short time to counteract with the target sites before parasites invasion to red blood cells. The most widely studied merozoite surface protein 1 (MSP1) is polymorphic and has a complicated folding pattern. *Plasmodium falciparum* MSP1 has a molecular weight of (200kDa) and is processed into a complex of polypeptides on the merozoite surface, with an 82kDa N-terminal polypeptide and 30kDa and 38kDa central regions, along with the 42kDa C-terminal region (MSP1 42). At the time of RBC invasion, MSP1-42 is processed, by proteolytic cleavage, into a 33kDa fragment (MSP1 33) and shed from the parasite with the rest of the MSP1 complex, and a C-terminal 19kDa fragment (MSP1-19). Only the C-terminal MSP1-19 portion remains on the surface and enters into the infected RBCs, this processing is suggested to be a critical step in the successful attack of parasites to RBCs (5).

The presence of glycosylation on the surface proteins of merozoites is still controversial, with studies showing different results, in particular on the Msp1 19kDa which is a vaccine candidate (6, 7). The extended range of cysteine-rich, glycan-recognizing proteins at all stages of the parasite lifecycle play essential roles in cell attachment and invasion (3). Carbohydrates (with terminal α-D-galactosyl residues) attached to malaria parasite proteins are suitable antigenic determinants and that they are recognized by the host's immune system (8). It is possible that the recombinant antigens used in the vaccines lacked glycosylation, which could be responsible for their failure in eliciting immunity. The presence of N and O-glycans on the MSP1 antigen is still controversial though some workers have shown its presence in the schizont and trophozoite stages.

In this study, glycosylation was investigated in the native form of 19kDa C-terminal of the MSP1 antigen in the ring stage using lectin affinity kits for detection of total and O-glycans along with bioinformatic work to verify the results.

## Materials and Methods

The study was carried out at the Biochemistry Department of Pasteur Institute of Iran in the years 2015–1016. *Plasmodium falciparum* 3D7 was cultured by the method of Trager and Jensen (9) using 7ml RPMI (GIBCO, In Vitrogen AB, Sweden) medium containing 5% hematocrit and 10% human AB negative serum with 0.1% gentamycin in a mixture of (92% N2, 5 % CO2, 3% O2) in a 25ml flask. The medium was changed every 48h, and the growth of the parasite was monitored by examination of Giemsa-stained smears under an oil-immersion objective lens.

Synchronization of parasites: An equal volume of 5% Sorbitol (Sigma-Aldrich, St Louis, Missouri, United States) was added to the parasite pellet followed by incubation for 10min at room temperature. The cultures were centrifuged and washed twice with RPMI and diluted to 5% hematocrit (10).

All reagents used were Sigma-Aldrich unless stated otherwise.

The collection of ring-stage parasites: Free parasites were obtained by adding 40 times the volume of 0.15% saponin at 4 °C for 30min. The cells were centrifuged at 1300rpm at 4 °C for 20 min. The pellet was washed with PBS and centrifuged at 4000rpm 3 times for 1min and stored at −20 °C.

Large scale production of parasites: The method of Radfar et al. (11) was adopted in which about 10% parasitemia was transferred to a 75ml flask with 1% hematocrit with daily changes of medium. When the parasites reached 20%, they were transferred to a 150ml flask with 1% hematocrit followed by synchronization described above. Daily changes of the medium were carried out until the parasites reached 60 %. Then, the parasites were harvested by centrifugation at 1500rpm for 7min and washed with sterile PBS.

The antigen was purified by isopycnic ultra-centrifugation using 500μl of the pellets mixed with TKM (50mM Tris-HCl containing 25mM KCl, 5mM MgCl2, 1mM EDTA containing 5% Triton-X, 0.01% 36% sucrose and protease inhibitors) to solubilize the parasite membrane (12) and centrifuged at 110,000xg for 16h after which the supernatant and the precipitate were both dialyzed separately to remove sucrose (13). Both fractions were analyzed by SDS-PAGE using 12% reducing gel with 125×145×1mm dimension by Laemmle's method (1970). The upper fraction contained the C-19 terminal of MSP1, and this was named as “parasite extract.”

Further purification was performed using preparative sodium dodecyl sulfate-polyacrylamide gel electrophoresis (SDS-PAGE) 125× 145×5mm using negative staining. Strips were cut at the 19KDa and the protein electro-eluted with a dialysis bag in Tris-glycine buffer pH 7.5 using 100 volts and 30mAmps overnight at 4 °C (14).

SDS-PAGE Staining methods:

- Silver staining was carried out using the method of Blum et al. (15).
- Coomassie blue R (CBB-R250) staining was carried out using 0.1% dye in 10% acetic acid, 40% methanol and 0.1% CBB-R250.
- Periodic acid Schiff's (PAS): staining was used to distinguish polysaccharides using method of Konat et al. (16).
- Reversible negative staining was carried out on preparative gels using imidazole zinc staining by the method of Castellanos et al. (17).

Immunoblotting: was carried out using (18) with the anti-C-terminal MSP1 *P. falciparum* 3D7 antibody prepared in rabbit, kindly donated by National Institute of Immunology, New Delhi, India

Glycoprotein analysis: was performed on the purified C-terminal fraction using O-glycan glycoprotein kit and total glycan glycoprotein kit (Qiagen, India Ltd.) (19). Briefly, for O-glycan kit, two affinity lectin columns AIL (javelin which is the jacalin lectin from *Artocarpus integrin folia* the jackfruit tree which attaches sialic acid and PNA from Peanut agglutinin which binds O-galactose units) and then eluted with their respective eluting buffers and the eluted proteins analyzed on SDS-PAGE using silver staining. For total glycoproteins, mannose-binding lectins and sialic glycoproteins were bound on concanavalin A and wheat germ agglutinin lectin columns and then eluted with respective eluting buffers. The eluted proteins were then analyzed by SDS-PAGE and silver staining.

The bioinformatic analysis was performed using C-terminal MSP1 genes: Different C- terminal partial cds were checked for glycosylation sites using EXPASY software (20). Genes of C- terminal MSP1 found in wild-type Iranian *P. falciparum* isolates registered earlier by the authors in the Gene Bank: ABQ52496, ABQ 52497, ABQ52498, ABQ57306, ABQ52495, ABM54034, BAA77608, (21) were used to detect predicted N and O-glycosylation (20).

## Results

**Fig. 1.**
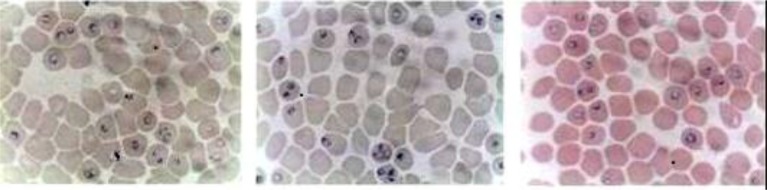
Synchronized large scale preparation of ring-stage *P. falciparum*

**Fig. 2.**
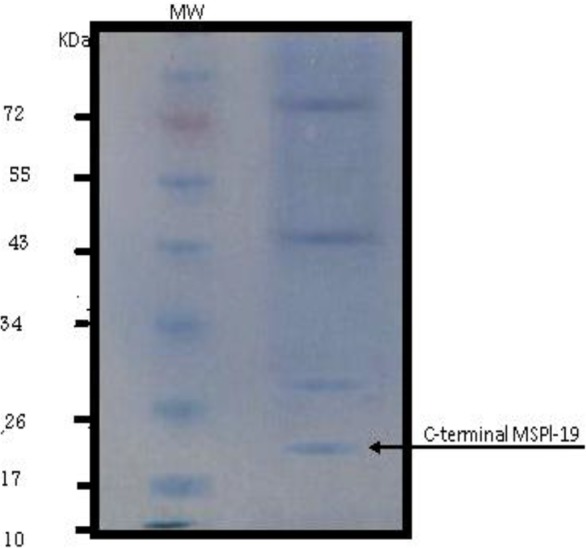
Coomassie blue staining of SDS-PAGE of parasite extract. MW is the molecular weight marker, and the right lane is *P. falciparum* “parasite extract”

**Fig. 3.**
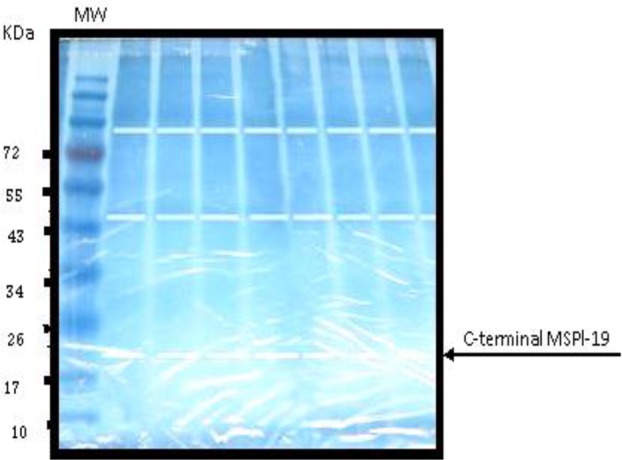
Preparative negative imidazole staining of SDS-PAGE of *P. falciparum* “parasite extract”. MW is molecular weight marker

**Fig. 4.**
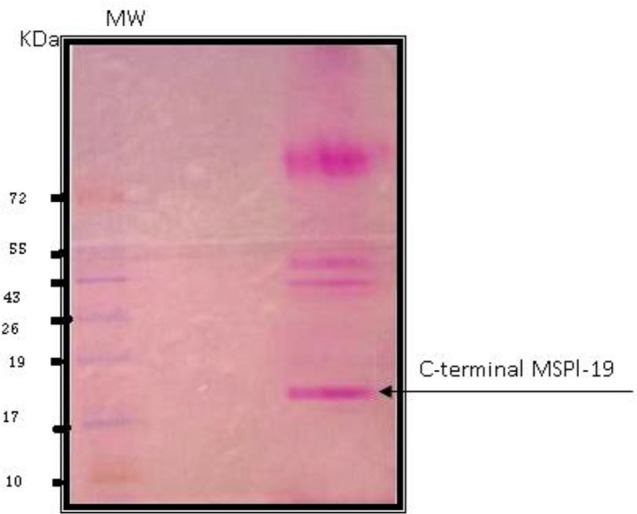
Glycoprotein Schiff's staining of SDS PAGE of *P. falciparum* “parasite extract”. MW is molecular weight markers

**Fig. 5.**
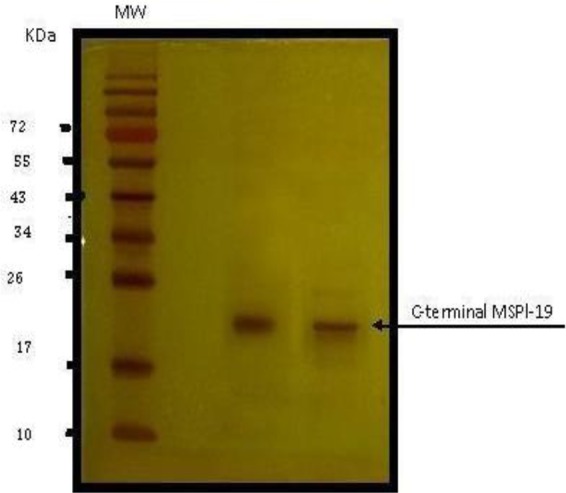
Silver-staining of SDS-PAGE of *P. falciparum* of purified C-19 kDa antigen. MW is molecular weight markers

**Fig. 6.**
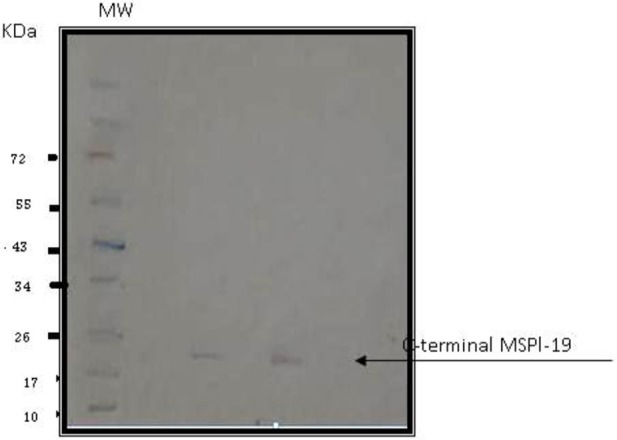
Immunoblotting of purified C-terminal 19kDa antigen using anti-C-terminal monoclonal antibody raised in rabbits. MW is molecular weight markers

**Fig. 7.**
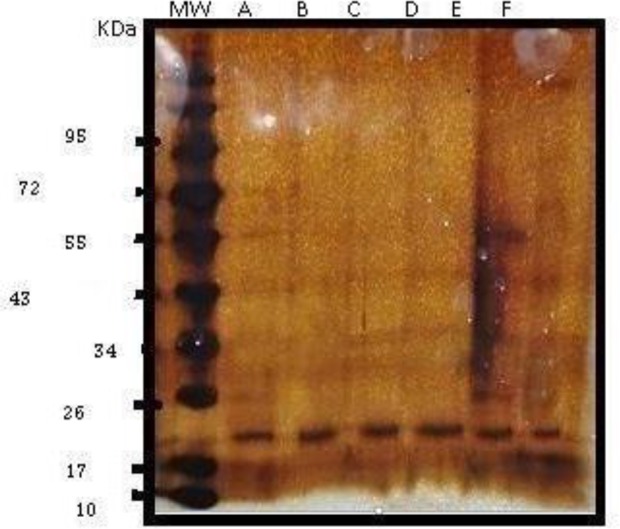
Silver staining of SDS-PAGE of purified antigen from O-proteome kit. MW is molecular weight markers. Lane A purified antigen C-terminal 19kDa, lane B Glycoproteins containing galactose and galactosamine eluted from PNA Kit, lane C containing N-acetyl neuraminic, galactose and galactosamine eluted from AIL column

Large scale preparation of parasites: A large number of ring-stage parasites were grown (Fig. 1). The SDS-PAGE analysis of parasite extract exhibited proteins stained by Coomassie blue staining in (Fig. 2). Preparative gels are used for cutting out the pure native MSP1-19kDa C-terminal antigen (Fig. 3) by reversible negative staining. Glycoproteins are exhibited in the MSP-1 antigen using Schiff's staining (Fig. 4). SDS analysis of pure native C-terminal 19kDa antigen: shows a single band with silver staining (Fig. 5). Immuno-blotting showed a single band with monoclonal anti-19kDa C-terminal antibody (Fig. 6). Silver staining for glycoproteins detected O-glycans in sialic acid and O-mannose binding units (Fig. 7) by the O-glycoprotein kit.

**Fig. 8.**
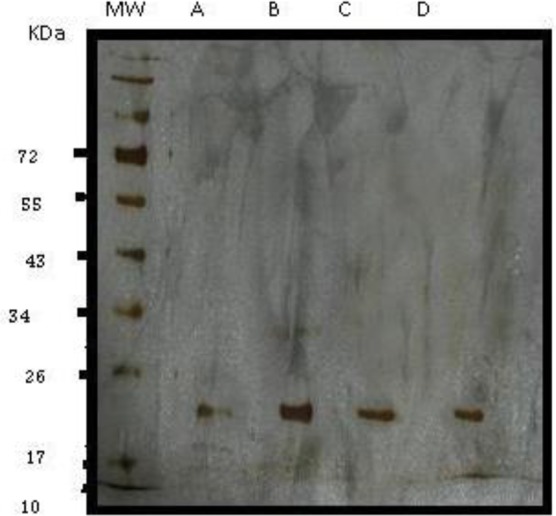
Silver staining of SDS-PAGE of purified antigen from total Q proteome kit, MW is molecular weight markers. Lane A purified antigen C-terminal 19kDa, lane B glycoproteins containing N-acetylglucosamine and sialic acid eluted from WGA column; lane C Mannose-containing glycoproteins eluted from Con A columns; lane D N-acetyl glucosamine and sialic acid glycoproteins eluted from WGA column

N-glycans comprising of complex bi-antennary and tri-antennary types, including sialic acid, were identified in the C terminal region of MSP1 19KDa (Fig. 8).

**Fig. 9.**
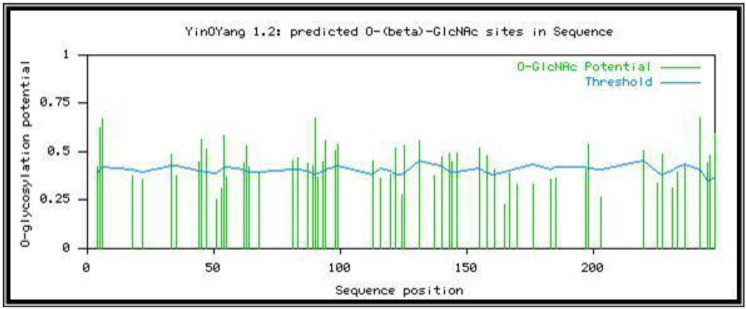
EXPASY file graphs showing 62 probable O-glycosylation sites of *P. falciparum* C-19kDa C-terminal MSP1 antigen and 5 potential N-glycan sites on the 250 base sequence

**Fig. 10.**
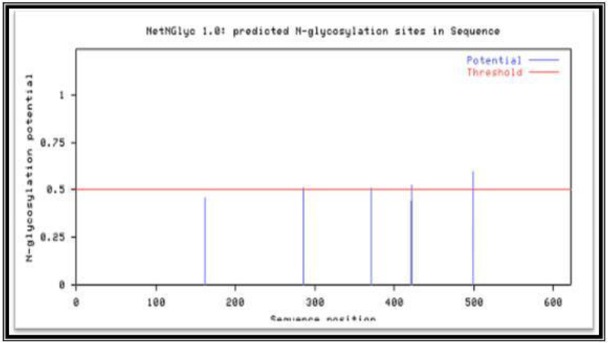
EXPASY file graphs showed five probable N-glycosylation sites of *P. falciparum* C-19kDa C-terminal MSP1 antigen

Bioinformatic analysis: The Expasy bioinformatic studies show the presence of 62 probable O-glycosylation sites (Fig. 9) and five potential N-glycan sites (Fig. 10) on the 250 base sequence.

## Discussion

Numerous researchers on glycobiology of *P. falciparum* have reported contradictory findings of the occurrence of glycoproteins on the *Plasmodium* surface proteins, especially MSP1 and merozoite surface protein 2 (MSP2). Currently, no reported of glycans on the C-terminal 19kDa MSP1 antigen. However, our studies have shown the presence of both N and O lectin binding glycans on the 19kDa C-terminal of MSP1. The N-glycans comprise asparagine binding N-acetylglucosamine including those bound to sialic acids. O-glycans are serine/threonine binding sialic acids with N-acetylgalactosamine and O-galactose units.

Neither MSP1 nor MSP2 shows an affinity for lectin binding or sensitivity to PNGase F, suggesting the absence of N-linked glycans (7). However, after the expression of MSP1 in mammalian cells (22) and the baculovirus system (23), the proteins are *N*-glycosylated. Glycosylation in the genus *Plasmodium* is mostly active in the biosynthesis of glycol phosphatidylinositol moiety (GPIs) attached to the C-termini of the proteins to anchor them to the bilipid bilayers (24). The MSP1s as their name denotes is on the surface and are closely associated with the GPIs. Comparative stoichiometric analysis has shown that two-thirds of all GPI-anchored proteins are associated with MSP1 and MSP2. Thirty GPI-anchored proteins are predicted to be expressed, and the existence of some potential N-glycosylation sites are shown in both proteins (25).

The availability of the *P. falciparum* genome has assisted research on the enzymes involved in N-glycosylation and even though the strain lacks most of the enzymes participating in the assembly of N-glycans which participates in a secondary loss of asparagine-linked glycosyltransferases enzymes for asparagine-linked glycans. The presence of two enzymes Alg7 and Alg14 responsible for the synthesis of dolicholpyro phosphoryl-oligosaccharides have been observed (26–28). The type of N-glycans produced in *P. falciparum* are shorter than their mammalian counterparts and transferred to glucosamine-labeled proteins and bind the corresponding lectin *G. simplicifolia* (29). As *P. falciparum* merozoite maturation takes place within an intraerythrocytic network of modified (parasitophorous vacuolar membrane) and newly made (tubo-vesicular network) membranes (30), it is likely that parasite surface proteins also comprise substrates for carbohydrate-modifying enzymes of the erythrocyte. There are reports of the absence of N-glycosylation machinery in *P. falciparum* parasites (30) even though N-linked carbohydrates have also been described in alliance with asparagines on MSP1 (31). However, our results showed the presence of lectin binding N-glycans on the C19 terminal of native MSP1 antigen by the total glycoprotein kit. We have confirmed the earlier studies that N-glycosylation is seen in the native antigen. The Expasy bioinformatic studies show the presence of some probable N-glycan binding sites. The partial Cds of the C-19kDa MSP1 genes (EF563844.1, EF563845.1, EF563846.1 (21) of a wild strain isolated from patients revealed the presence of asparagine residues corroborating the results of the Expasy website.

We have confirmed the presence of O-glycans in native 19kDa C-terminal of MSP1 by their binding to AIL and PNA lectin columns which exhibit the presence of sialic acid with N-acetylgalactosamine and O-galactose units. Our results confirm that of Nasir-Ud-din 1991 for MSP1 indicating the presence of glucosamine and galactose in the protein (31), and also Gilson (30) treated galactose, fucose and glucosamine-radiolabeled proteins with PNGase-F to remove possible N-glycans, and purified the resulting material by gel filtration and subjected the void volume fraction to alkaline β-elimination. About half of the radioactivity was shifted to the monosaccharide volume, proposing the discharge of O-glycans. They also characterized O-glycosylation reactions using exogenous galactosylases to identify O-GlcNAc in proteins. Other studies (25, 32) reported no O-glycosylation in the blood during the asexual stage of the parasite. However, our work on the asexual stage shows the presence of O-glycosylation which confirms another the work (13). It was used immunoprecipitation to show the C-terminal fragment of MSP1 contains O-glycosylation sites. Moreover, the whole protein, as well as both N and C-terminal MSP-1 fragments, could be exo-galactosylated. Recently, the presence of OGT (genes for O-GlcNAcylation) has been investigated using anti-OGT antibodies and these studies have proposed the presence of OGlcNAcylation in *P. falciparum* (33) supporting earlier observations of GlcNAcβ-Ser/ Thr in MSP2 (6). In our study, the five different 19 kDa C-terminal MSP1 sequences obtained from various *P. falciparum* strains showed the presence of both threonine and serine in the 19kDa C-terminal sequence, needed for glycosylation. Hoessli and co-workers (13) have worked on the schizont and late trophozoite stage, but we have shown the presence of N and O-glycans in the ring stage of the C-19kDa antigen. The Expasy bioinformatics site showed the probable presence of more than 60 O-glycosylation sites for the seven genes isolated from Iranian strains verifying our experimental work.

The life cycle of the *P. falciparum* comprises of the ring stage in the RBC changed to trophozoites and then finally to schizont stage. The schizonts release the merozoites, which once again infect the red blood cells. We have shown N and O-glycans in the ring stage of the native MSP1 C-19kDa antigen. As earlier workers have demonstrated its presence in the other two stages, it is likely that contrary to all other speculations, the glycosylation of this antigen in its native form is seen in all the stages.

The purity of our antigen was quite high as it is was purified by both ultracentrifugation and preparative SDS-gel electrophoresis and gave a single band on immunoblotting using the specific monoclonal antibody against C-terminal 19 kDa. Specific Schiff's staining for carbohydrates also shows the presence of glycoproteins in the C-terminal 19kDa antigen along with the Q proteome kit. Bioinformatics analysis too shows the presence of many likely O-glycoprotein sites on the C-terminal antigen and also N-terminal binding ones.

## Conclusion

Both N and O-glycan antigens are present in the native form of the C-terminal of 19kDa antigen in the ring stage. It is possible that the lack of elicitation of protection by recombinant MSP1 antigen could be due to their pre-requisition of glycosylation. Further research on recombinant antigens should concentrate on their glycosylation if they are to be used as vaccine candidates.

The presence of N and O-glycoproteins were detected by Q proteome kit on the native 19kDa C-terminal MSP1 purified from the ring stage of *P. falciparum*. Taking into account the data of earlier scientists that it is likely that MSP1 is glycosylated in all the stages of the parasite life cycle and incomplete protection elicited by recombinant MSP1 antigens is due to lack of N and O-glycoproteins.
